# The implications of clinical risk factors, CAR index, and compositional changes of immune cells on hyperprogressive disease in non-small cell lung cancer patients receiving immunotherapy

**DOI:** 10.1186/s12885-020-07727-y

**Published:** 2021-01-05

**Authors:** Seo Ree Kim, Sang Hoon Chun, Joo Ri Kim, Sang-Yeob Kim, Jun Young Seo, Chan Kwon Jung, Bo-Mi Gil, Jeong-Oh Kim, Yoon Ho Ko, In Sook Woo, Byoung Yong Shim, Sook-Hee Hong, Jin Hyoung Kang

**Affiliations:** 1 Division of Medical Oncology, Department of Internal Medicine, Bucheon St. Mary’s Hospital, College of Medicine, The Catholic University of Korea, Seoul, Republic of Korea; 2 Division of Medical Oncology, Department of Internal Medicine, Seoul St. Mary’s Hospital, College of Medicine, The Catholic University of Korea, 222, Banpo-daero, Seocho-gu, Seoul, 06591 Republic of Korea; 3 Asan Institute for Life Sciences, Asan Medical Center, University of Ulsan College of Medicine, Seoul, Republic of Korea; 4 Department of Convergence Medicine, Asan Medical Center, University of Ulsan College of Medicine, Seoul, Republic of Korea; 5 Department of Hospital Pathology, College of Medicine, the Catholic University of Korea, Seoul, Republic of Korea; 6 Department of Radiology, Bucheon St. Mary’s Hospital, College of Medicine, The Catholic University of Korea, Seoul, Republic of Korea; 7 Cancer Research Institute, College of Medicine, The Catholic University of Korea, Seoul, Republic of Korea; 8 Division of Medical Oncology, Department of Internal Medicine, Uijeongbu St. Mary’s Hospital, College of Medicine, The Catholic University of Korea, Seoul, Republic of Korea; 9 Division of Medical Oncology, Department of Internal Medicine, Yeouido St. Mary’s Hospital, College of Medicine, The Catholic University of Korea, Seoul, Republic of Korea; 10 Division of Medical Oncology, Department of Internal Medicine, St. Vincent’s Hospital, College of Medicine, The Catholic University of Korea, Seoul, Republic of Korea; 11 Laboratory of Medical Oncology, Cancer Research Institute, College of Medicine, The Catholic University of Korea, Seoul, Republic of Korea

**Keywords:** Non-small cell lung cancer (NSCLC), Immune checkpoint blockades (ICBs), M2 macrophage, Tumor-infiltrating lymphocyte (TIL), Tumor microenvironment

## Abstract

**Background:**

Immune checkpoint blockades (ICBs) are characterized by a durable clinical response and better tolerability in patients with a variety of advanced solid tumors. However, we not infrequently encounter patients with hyperprogressive disease (HPD) exhibiting paradoxically accelerated tumor growth with poor clinical outcomes. This study aimed to investigate implications of clinical factors and immune cell composition on different tumor responses to immunotherapy in patients with non-small cell lung cancer (NSCLC).

**Methods:**

This study evaluated 231 NSCLC patients receiving ICBs between January 2014 and May 2018. HPD was defined as a > 2-fold tumor growth kinetics ratio during ICB therapy and time-to-treatment failure of ≤2 months. We analyzed clinical data, imaging studies, periodic serologic indexes, and immune cell compositions in tumors and stromata using multiplex immunohistochemistry.

**Results:**

Of 231 NSCLC patients, PR/CR and SD were observed in 50 (21.6%) and 79 (34.2%) patients, respectively and 26 (11.3%) patients met the criteria for HPD. Median overall survival in poor response groups (HPD and non-HPD PD) was extremely shorter than disease-controlled group (SD and PR/CR) (5.5 and 6.1 months vs. 16.2 and 18.3 months, respectively, *P* = 0.000). In multivariate analysis, HPD were significantly associated with heavy smoker (*p* = 0.0072), PD-L1 expression ≤1% (*p* = 0.0355), and number of metastatic site ≥3 (*p* = 0.0297). Among the serologic indexes including NLR, PLR, CAR, and LDH, only CAR had constantly significant correlations with HPD at the beginning of prior treatment and immunotherapy, and at the 1st tumor assessment. The number of CD4+ effector T cells and CD8+ cytotoxic T cells, and CD8+/PD-1+ tumor-infiltrating lymphocytes (TIL) tended to be smaller, especially in stromata of HPD group. More M2-type macrophages expressing CD14, CD68 and CD163 in the stromal area and markedly fewer CD56+ NK cells in the intratumoral area were observed in HPD group.

**Conclusions:**

Our study suggests that not only clinical factors including heavy smoker, very low PD-L1 expression, multiple metastasis, and CAR index, but also fewer CD8+/PD-1+ TIL and more M2 macrophages in the tumor microenvironment are significantly associated with the occurrence of HPD in the patients with advanced/metastatic NSCLC receiving immunotherapy.

**Supplementary Information:**

The online version contains supplementary material available at 10.1186/s12885-020-07727-y.

## Background

Immune checkpoint blockades (ICBs), which blocks CTLA-4, PD-1 or PD-L1, exert anti-tumor activities through re-invigorating exhausted T-lymphocytes [1–3]. Clinical excitement regarding these ICBs has resulted from their different advantages, including the unprecedented number of durable clinical responses and better tolerability among patients with a variety of advanced cancer types [4–6]. However, in some cases, patients not infrequently exhibit a paradoxically accelerated tumor growth with poor outcome; such cases are designated as hyperprogressive disease (HPD). Even though the definition of HPD and predisposing factors somewhat differ depending on the source, one consistent finding is that the growth kinetics at first tumor assessment is more than double compared to that at the beginning of immunotherapy [7, 8].

The tumor is surrounded by a complex and heterogeneous tumor microenvironment (TME), comprised of several types of immune cells, fibroblasts, and a tumor-specific extracellular matrix [3, 9, 10]. There is abundant evidence that tumor cells and the TME constantly interact to modulate tumor growth [4, 9]. Regarding tumor-associated factors, certain genetic aberrations such as *MDM2/4* amplification and *KRAS-SKT11* mutation, have been reported to be associated with HPD. In addition, down-regulated T cell signatures and a high density of M2-type macrophages and myeloid derived suppressor cells, which exist in TME, have shown negative impacts on immunotherapy [10–13].

Earlier studies demonstrated that tumor-infiltrating lymphocytes (TILs) were strongly associated with local PD-L1 expression in the tumor biopsies of melanoma patients [14]. To suppress local effector T-cell function, tumor cells upregulate PD-L1 expression in response to interferon-γ released by TILs as an adaptive immune-resistance mechanism [2, 7, 15, 16]. In addition, increased levels of CD3 and CD8+ TILs were associated with better outcome in a large series of patients with non-small cell lung cancer (NSCLC) [15]. Moreover, PD-L1 can also be expressed constitutively on cancer cells through poorly characterized oncogenic signaling pathways [17]. Indeed, PD-L1 expression is observed in various cancers including lung, melanoma, breast, kidney cancer, and Hodgkin lymphoma, and it is widely accepted as key biomarker for predicting clinical response to anti–PD-1/PD-L1 therapy [5, 18, 19].

On the basis of PD-L1 status and presence or absence of TILs, malignant disease can be classified into four groups: type I (PD-L1 positive with TILs driving adaptive immune resistance), type II (PD-L1 negative with no TIL indicating immune ignorance), type III (PD-L1 positive with no TIL indicating intrinsic induction), and type IV (PD-L1 negative with TIL indicating the role of other suppressor(s) in promoting immune tolerance) [2, 20]. In addition to natural killer (NK) cells and dendritic cells, cancer associated fibroblasts, which regulate the dynamic process of M2 transformation, can affect the response to cancer immunotherapy [10]. Meanwhile, inflammatory reaction indices in the peripheral blood do not directly reflect the local immune responses occurring at tumor; nonetheless, the systemic indices closely connected with the tumor response to immunotherapy [21, 22].

Accordingly, we hypothesized that there may be meaningful distinctions in clinical features, serologic markers, and compositional changes of immune cells among patient groups displaying different tumor response to immunotherapy. We therefore conducted a retrospective analysis of clinical data, periodically monitored serological indices, and quantitatively analyzed immune cell compositions of the intratumoral and stromal regions.

## Methods

### Study population and design

Data were retrospectively collected from all consecutive eligible patients with advanced NSCLC who were treated with ICBs between January 2014 and May 2018, at five St. Mary’s Hospitals in Seoul, Suwon, Uijeongbu, Bucheon, and Yeouido, Korea. This study was approved by the Institutional Review Board of Catholic Medical Center [KC18SESI0440]. ICBs including nivolumab, pembrolizumab, avelumab, atezolizumab, or durvalumab were prescribed under coverage by health insurance or an early access program. We excluded patients who were lost to follow-up while showing a favorable response to ICBs or who did not have information available regarding the previous treatment.

Clinical data included age at diagnosis, sex, primary tumor location, TNM stage at diagnosis, number of prior systemic treatments, best tumor response during immunotherapy, baseline and post-immunotherapy imaging, patterns of recurrence, and location of distant metastases. Patients were divided into four groups: HPD, non-HPD progressive disease (non-HPD PD), stable disease (SD), and partial/complete response (PR/CR) displaying different tumor response to immunotherapy. We recorded time-series laboratory data including serum C-reactive protein, erythrocyte sedimentation rate, albumin, lactate dehydrogenase (LDH), and white blood cell count immediately before starting treatment, at the beginning of immunotherapy, and at the first tumor response assessment, i.e., 6–8 weeks after initiation of immunotherapy. The neutrophil-to-lymphocyte ratio (NLR) was defined as the absolute neutrophil count divided by absolute lymphocyte count, and the platelet-to-lymphocyte ratio (PLR) was defined as platelet count divided by the lymphocyte counts. The C-reactive protein-to-albumin ratio (CAR) was calculated by dividing the C-reactive proteins level by the albumin level.

### Tumor growth kinetics

Radiological changes were evaluated based on the Response Evaluation Criteria in Solid Tumors version 1.1 (RECIST ver. 1.1) [23] and iRECIST [24]. We defined HPD as having (1) a tumor growth kinetics ratio (TGKr) exceeding the tumor growth rate by two-fold between the reference period (before immunotherapy) and the experimental periods during anti–PD-1/PD-L1 therapy and (2) a time-to-treatment failure (TTF) less than 2 months [7, 13, 25, 26]. We reviewed all pre- and post-immunotherapy images and determined the two points for determining tumor growth kinetics (i.e., before starting immunotherapy [TGK_PRE_] and after immunotherapy [TGK_POST_]) [12, 13, 21]. T_PRE_, T_0_, and T_POST_ denote the time of the reference period’s baseline, experimental period’s baseline, and the experimental period’s first-post imaging, respectively. S_PRE_, S_0_, and S_POST_ denote the sum of the largest diameter of target lesions at the reference period’s baseline, experimental period’s baseline, and first follow-up image of the experimental periods, respectively. TGK_PRE_ was defined as the difference in the sum of the largest diameters of the target lesions per unit of time between the reference period and experimental baseline imaging: (S_0_S_PRE_)/(T_0_T_PRE_). Similarly, TGK_POST_ was defined as (S_0_S_POST_)/(T_0_T_POST_). TGKr was defined as the ratio of TGK_POST_ to TGK_PRE_. TGKr > 1 indicated tumor growth acceleration, whereas 0 < TGKr ≤1 and TGKr ≤0 indicated tumor deceleration and tumor shrinkage, respectively [13, 25–27].

### Assessment of PD-L1 expression level using immunohistochemistry

We used archival tumor tissues obtained by core needle biopsy or excisional biopsy at the initial diagnosis. PD-L1 expression is widely used as a key predictive biomarker for PD-1/PD-L1 blockade and has been approved as a companion diagnostic test for pembrolizumab (Kytruda®; Merck, Kenilworth, NJ, USA). PD-L1 expression was assessed using immunohistochemistry (IHC) in formalin-fixed paraffin-embedded tumor tissue using the PD-L1 IHC 22C3 pharmDx assay (Dako, Santa Clara, CA, USA) at a hospital pathology laboratory. These data were determined by means of a Combined Positive Score, which includes the number of PD-L1 positive cells (tumor cells, lymphocytes, macrophages) divided by the total number of viable tumor cells, multiplied by 100.

### Analysis of immune cell composition using multiplex IHC

In order to examine the TME, we used a quantitative multispectral imaging method using the Opal Multiplex IHC kit (Perkin-Elmer, Waltham, MA, USA) and Vectra automated quantitative pathology imaging system (Perkin-Elmer). Multiplex IHC staining for immune cells and antagonists of the PD-1/PD-L1 pathway was performed using a Leica Bond Rx™ Automated Stainer (Leica Biosystems, Newcastle, UK). We analyzed scanned images using inForm image analysis software (Perkin-Elmer) and TIBCO Spotfire software (TIBCO, Palo Alto, CA, USA).

We analyzed differences in the immune composition of the TME using multiplex IHC. T cell markers, including CD4, CD8, FOXP3, CD45RO, and CD3 were placed on panel 1, and co-inhibitory signal markers including TIM3, LAG3, PD-1, and PD-L1 were placed on panel 2. We also examined the degree of penetration of CD14, CD68, CD163, and CD206 as macrophage markers on panel 3 as well as CD11c as a myeloid-derived cell marker, CD16, CD56, CD86, and CD103 as NK cell and dendritic cell markers on panel 4.

### Statistical analysis

Independent t-test and Chi-squared test were used to analyze differences in baseline patients’ characteristics and clinicopathological factors. In the multivariate analyses, logistic regression was performed to examine the risk factors of HPD. Overall survival was estimated using the Kaplan-Meier method and it was calculated from the start of ICB administration until the date of death or last follow-up. All statistical analyses were performed using SAS program (version 9.4;SAS Institute Inc., Cary, NC, USA). Spider plots, scatter plots, and Kaplan-Meier survival curve were generated using GraphPad Prism 8.0 (GraphPad Software, Inc., San Diego, CA, USA). In all statistical analyses, a two-sided *P* value of < 0.05 was considered statistically significant.

## Results

### Baseline clinical characteristics

Altogether 231 patients were included in the tumor growth kinetics analysis, with a mean age of 64.2 years, male sex composition of 74.9% and ex- or current smoker composition of 70.2%. Among the smokers, heavy smokers (≥20 packs per year) comprised 88.9%. Most of the patients were treated with ICBs as at least second line and 37 patients were heavily treated (≥ 4th line treatment). PR/CR and SD were achieved in 50 (21.6%) and 79 (34.2%) patients, respectively. Twenty patients (8.7%) did not have a response evaluation due to rapid progression with early death and were subsequently classified as the NE (non-evaluable) group (Supplementary Fig. 1). Of 82 patients exhibiting PD, 26 (31.7%) met the criteria for HPD (Supplementary Fig. 2). The baseline characteristics of all patients are listed in Supplementary Table 1, Supplementary Fig. 1 and 2.

### Correlation between clinical and pathological parameters and tumor response pattern

When compared with disease-controlled group (defined as SD, and PR/CR), HPD was markedly frequent in patients carrying oncogenic driver mutations (30.8%, *P* = 0.018). Furthermore, there were significant differences in age (*P* = 0.002), multiple metastatic sites (≥ 3) (*P* = 0.005), and number of prior treatment line (*P* = 0.139) between disease-controlled group and HPD group (Table 1). In 155 patients, PD-L1 expression level was different among the tumor response groups. PD-L1 expression in the HPD group tended to be lower compared to that of the disease-controlled group and HPD occurred more frequently in patients with very low PD-L1 expression (< 1%) (*P* = 0.003) (Supplementary Fig. 3). Additionally, very shortened overall survival times were observed in HPD and non-HPD PD group, when compared with SD and PR/CR groups (5.5 months and 6.1 months vs 16.2 months and 18.3 months, respectively, *P* = 0.000) (Supplementary Fig. 4).

**Table 1:** Patients’ clinical characteristics according to tumor response pattern (*n* = 155)^a^

Total patients	HPD (***n*** = 26)	SD/PR/CR (***n*** = 129)	***P*** value
**Age (year)**	58.96 ± 10.2	65.19 ± 9.1	0.002
**Gender, Female: Male**	3:23	35:94	0.092
**Smoking history (pack/y)**	29.3 ± 19.6	24.41 ± 21.6	0.284
< 20	5	51	0.049
≥ 20	21	78	
**Histologic subtype**			0.061
Adenocarcinoma	14	93	
Squamous cell carcinoma	11	34	
Others	1	2	
**Oncogenic driver mutations**			
Yes: No	8:18	16:113	0.018
*EGFR* mutation	5	7	
ALK, ROS1, and others	3	9	
**Number of metastatic sites**			0.005
< 3	17	113	
≥ 3	9	16	
**Prior treatment lines before ICBs**			0.139
< 3	21	117	
≥ 3	5	12	

Values are presented as mean ± standard deviation, ratio, or number

^a^Non-evaluable group and non-HPD PD group were excluded

*ALK* Anaplastic lymphoma kinase, *EGFR* epidermal growth factor receptor, *HPD* hyperprogressive disease, *Non-HPD PD* non-HPD progressive disease, *OS* overall survival, *PR/CR* partial/complete response, *SD* stable disease

### Changes of inflammation-related serologic markers in tumor response groups

Table 2 contains the associations of tumor response pattern with NLR, PLR, CAR, and LDH at serial time points. Among these serologic markers showing close connection with HPD at the time of first response evaluation, only CAR still had significant correlations at the beginning of immunotherapy and prior treatment.

**Table 2:** The changes of serologic markers at three clinical situations in HPD, SD, and PR/CR groups (*n* = 155)^a^

Serologic marker		HPD	SD	PR/CR	***P*** value
		(***n*** = 26)	(***n*** = 79)	(***n*** = 50)	
**At the beginning of prior treatment**					
NLR	< 5 vs. ≥ 5	17:9	58:21	39:11	0.341
PLR	< 150 vs. ≥ 150	8:18	33:46	16:34	0.835
CAR	< 0.5 vs. ≥ 0.5	12:14	63:16	40:10	**0.004**
LDH	< 400 vs. ≥ 400	9:17	30:49	17:33	0.706
**At the beginning of immunotherapy**					
NLR	< 5 vs. ≥ 5	17:9	65:14	39:11	0.203
PLR	< 150 vs. ≥ 150	8:18	33:46	22:28	0.265
CAR	< 0.5 vs. ≥ 0.5	10:16	57:22	32:18	**0.026**
LDH	< 400 vs. ≥ 400	10:16	26:53	18:32	0.581
**At 1st tumor response assessment (6–8 weeks after initiation of ICBs)**					
NLR	< 5 vs. ≥ 5	8:18	71:8	45:5	**0.000**
PLR	< 150 vs. ≥ 150	7:19	42:37	29:21	**0.004**
CAR	< 0.5 vs. ≥ 0.5	9:17	56:23	32:18	**0.001**
LDH	< 400 vs. ≥ 400	4:22	24:55	19:31	**0.043**

^a^ Non-evaluable group and Non-HPD PD group were excluded

*CAR* C-reactive protein-albumin ratio, *ICB* immune checkpoint blockades, *LDH* lactate dehydrogenase, *NLR* neutrophil-to-lymphocyte ratio, *PLR* platelet-to-lymphocyte ratio

### Risk factors for HPD by univariate and multivariate analyses

The results of logistic regression analyses of clinical factors associated with HPD are listed in Table 3. Because inflammation-related serologic indexes had mutual interference, they were excluded from a separate statistical analysis. Univariate analysis revealed that ECOG PS 2–3 (*p* = 0.0572), smoking ≥20 pack. Years (*p* = 0.0563), PD-L1 expression ≤1% (*p* = 0.0049), the presence of oncogenic driver mutation (*p* = 0.0227), and number of metastatic sites ≥3 (*p* = 0.0073) are risk factors for HPD. In multivariate analysis, heavy smoker (*p* = 0.0072), very low PD-L1 expression (*p* = 0.0355), and multiple metastatic sites were significantly associated with HPD. The inflammation-related serologic markers were associated with HPD by univariate and multivariate analyses (Supplementary Table 2).

**Table 3:** Clinical factors associated with HPD by univariate and multivariate analyses (n = 155)^a^

	Univariate		Multivariate	
	HR (95% CI)	***P***-value	HR (95% CI)	***P***-value
Age, ≥65 vs < 65	0.49 (0.20–1.18)	0.1115		
Gender, Female vs. Male	0.35 (0.10–1.24)	0.1039		
ECOG, 2 ~ 3 vs. 0 ~ 1	10.67 (0.93–122.33)	0.0572	6.39 (0.48–8575)	0.1613
Smoking (pack.year), ≥20 vs. < 20	2.75 (0.97–7.75)	0.0563	5.62 (1.59–19.78)	**0.0072**
PD-L1 expression > 1 vs ≤1	0.28 (0.12–0.68)	**0.0049**	0.35 (0.13–0.93)	**0.0355**
Oncogenic driver mutation, any one vs. no	3.14 (1.17–8.39)	**0.0227**	3.21 (0.97–10.60)	0.0552
EGFR mutation, positive vs. negative	2.32 (0.74–7.27)	0.1483		
No. of metastatic site, ≥3 vs. < 3	3.74 (1.43–9.79)	**0.0073**	3.53 (1.13–10.99)	**0.0297**
No. of treatment line before IO, ≥4 vs. < 4	2.30 (0.73–7.21)	0.1526		

^a^ Non evaluable group and Non-HPD PD group were excluded

*HPD* hyperprogressive disease, HR hazard ratio, ECOG European Cooperative Oncology Group, PD-L1 programmed death-ligand 1, *EGFR* epidermal growth factor receptor, IO immune oncology therapy

### Analysis of immune cell composition in the TME by multiplex IHC

**Fig. 1:**
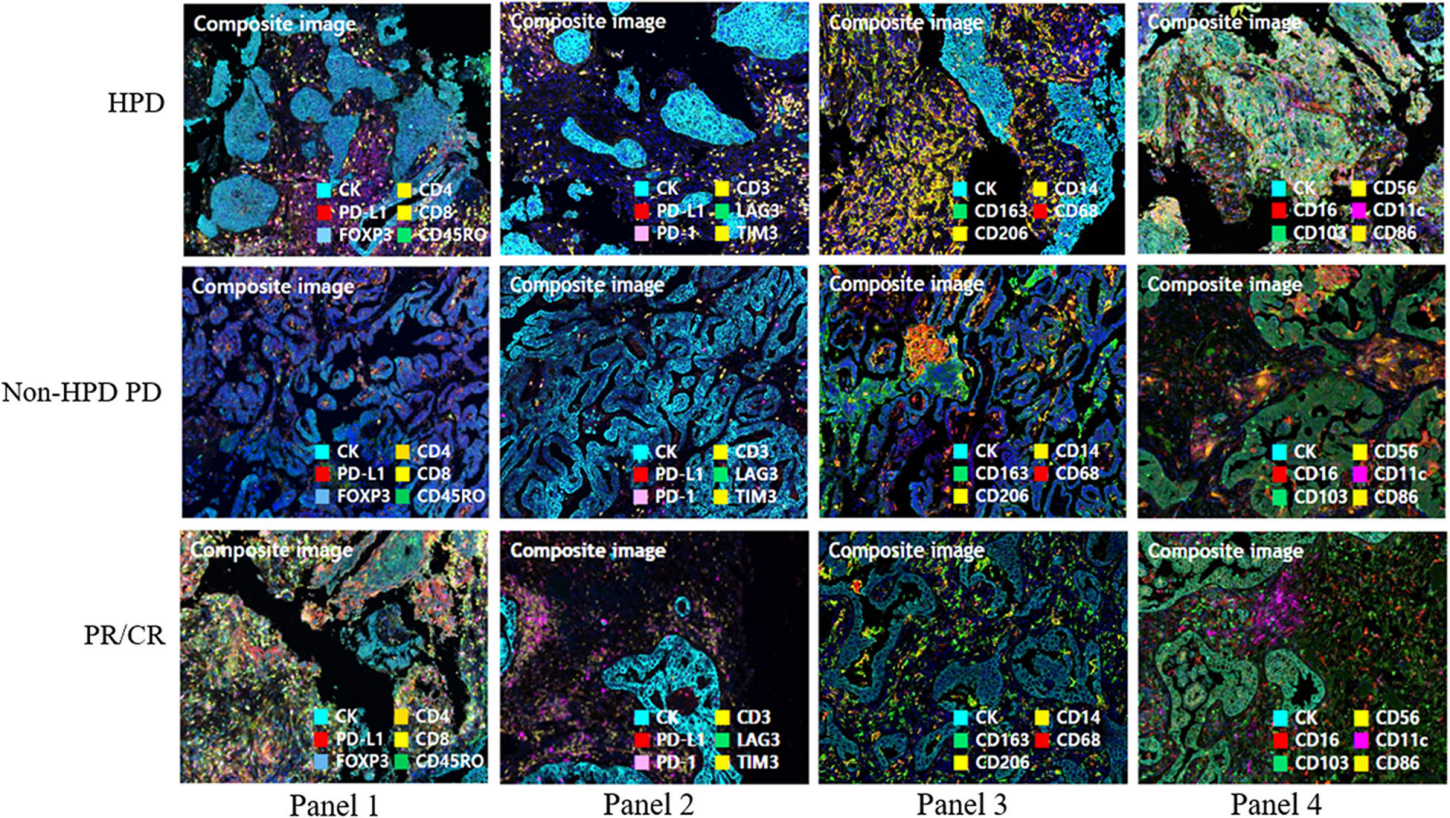
Multiplex immunohistochemistry for immune cell markers in HPD, non-HPD PD, and PR/CR group (*n* = 24) ^†^As explorative setting, some cases with available tissues in HPD, non-HPD PD and PR/CR group. *HPD* hyperprogressive disease, *NK* natural killer cells, *Non-HPD PD* non-HPD progressive disease

**Fig. 2:**
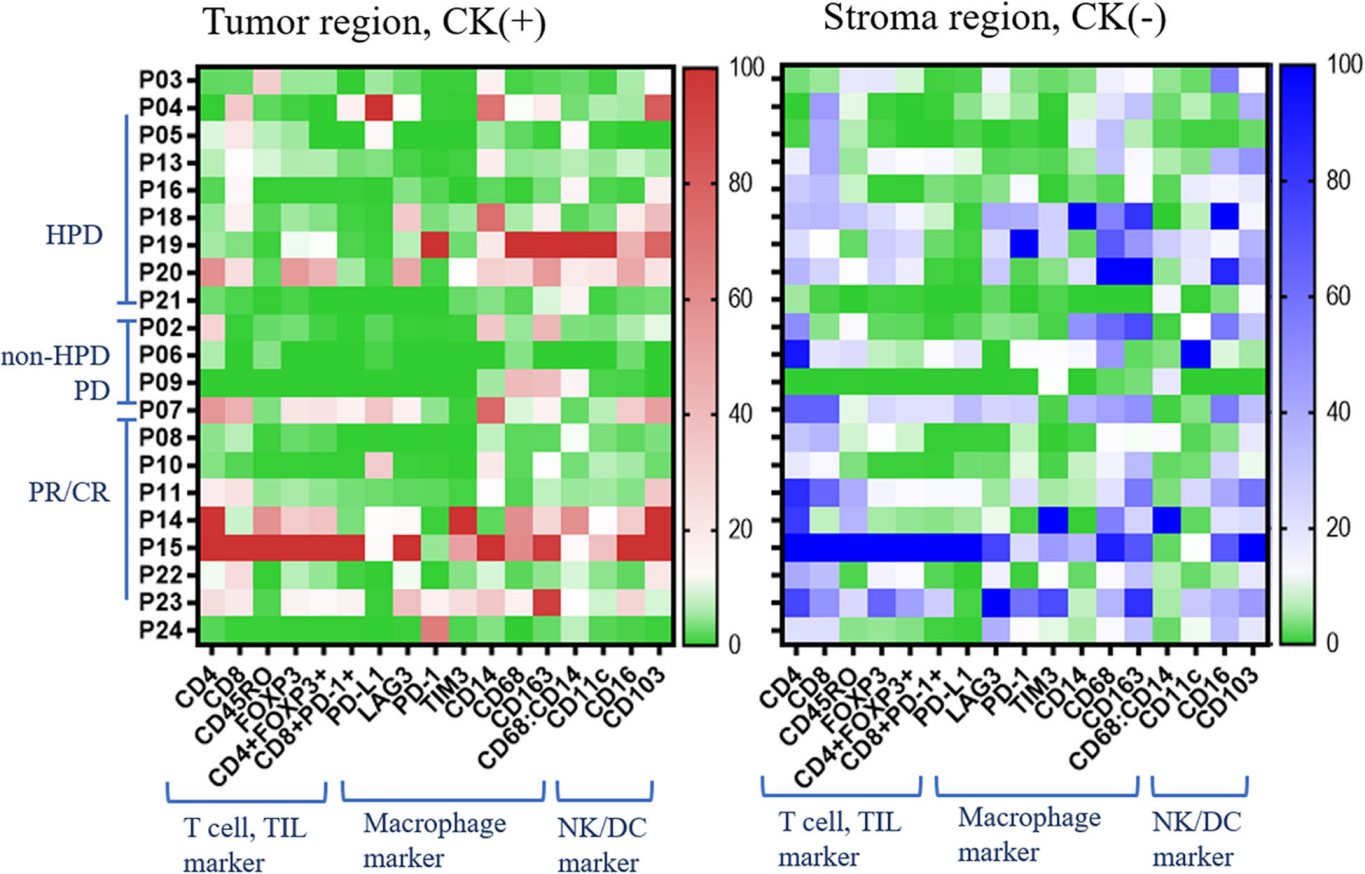
Multiplex immunohistochemistry panels displaying immune compositional changes in tumor microenvironment (*n* = 24)^†^ . ^†^As explorative setting, some cases with available tissues in HPD, non-HPD PD and PR/CR group. *HPD* hyperprogressive disease, *Non-HPD PD* non-HPD progressive disease, *PR/CR* partial response/complete response, *TIL* tumor infiltrating lymphocytes, *NK* natural killer cells, *DC* dendritic cells

**Fig. 3:**
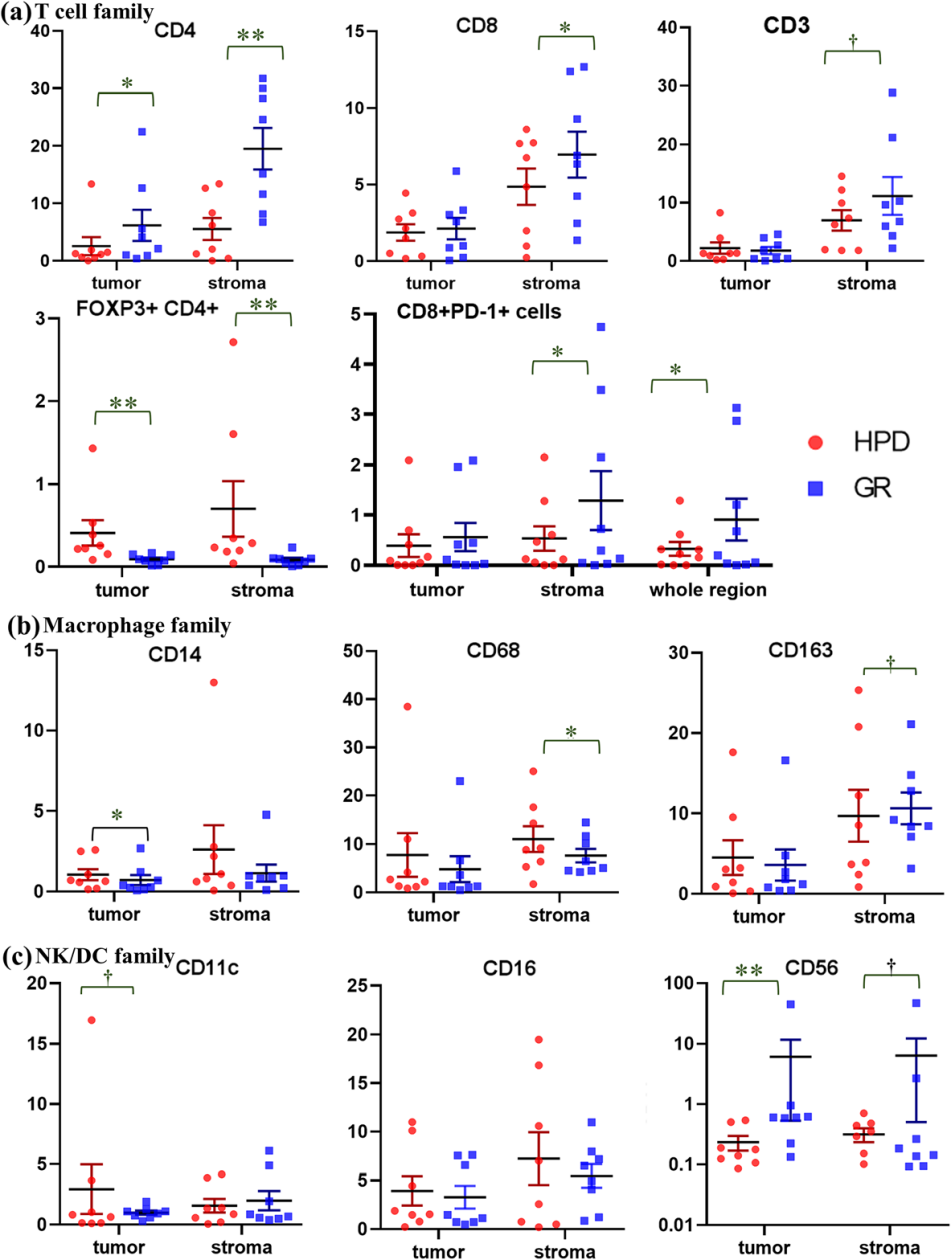
Immune cell composition in the tumor and stroma using Multiplex IHC. **a** The numbers of CD4+ and CD8+ lymphocytes are significantly smaller in the HPD group compared to those of the disease-controlled group. FOXP3 + CD4+ Treg cells are significantly larger in number in the HPD group. **b** CD68+ and CD163+ macrophages are more frequently observed in stroma of the HPD group, suggestive of M2 polarization. **c** CD11c + CAFs tend to increase and CD56+ NK cells are significantly reduced in the intra-tumoral area in the HPD group. *IHC* immunohistochemistry, *HPD* hyperprogressive disease, *NK* natural killer cells, *DC* dendritic cells. Statistical power: ** implies p value < 0.05, * implies *p* value < 0.5, ^†^ implies p value ≥0.5 but shows a clinical correlation

To understand the cross-talk between the tumor and its accompanying heterogeneous TME, we analyzed the expression of several types of immune cells using multiplex IHC as explorative setting, only some cases with many available tissues (Fig. 1). In the PR/CR group, the number of cells expressing T cell and TIL markers tended to be higher in the entire area, but especially in the stroma. In contrast, in the HPD group, cells expressing macrophage markers were markedly higher in both the tumor and stroma. There was a noticeable increase of M2 marker-positive cells in the stroma (Fig. 2). In the HPD group, there were fewer CD4+ effector T cells and CD8+ cytotoxic T cells (*P* <  0.010 and *P* <  0.382, respectively), whereas, there were significantly more regulatory T (Treg) cells co-expressing CD4+ and FOXP3+ in both the tumor and stroma (*P* <  0.003 and *P* <  0.015, respectively). CD8+/PD-1+ cells, a parameter for TIL activity, were significantly lower in HPD group than in the PR/CR group, and the degree of immune cell penetration into the tumor region did not increase (Fig. 3a). In addition, there were significantly more macrophages expressing CD14, CD68, and CD163 in the TME of the HPD group, implying a tendency for M2 polarization in HPD (Fig. 3b). In the HPD group, CD11c, one of the myeloid markers expressed by cancer-associated fibroblasts, tended to increase intratumorally, but CD103 (a marker of dendritic cells) and CD56 (a maker of NK cells) decreased (Figs. 2 and 3c). Interestingly, there was a reverse composition of CD56+ NK cells in the tumor and stromal regions between the PR/CR and HPD groups. Even though CD56+ NK cells in stromal area did not differ between the two groups, there were significantly more intratumoral CD56+ NK cells in PR/CR group (Fig. 3c).

## Discussion

Although immunotherapy can induce favorable and durable tumor responses in some patients, many patients experienced a poor prognosis with tumor flare-ups [5–7, 28]. Generally, HPD is defined as a TGKr exceeding 2 during anti–PD-1/PD-L1 therapy and a TTF of ≤2 months. The incidence of HPD is diverse, ranging between 4 and 29% [7, 13, 25–27]. This study analyzed associations of clinical and serological parameters with the tumor response pattern and evaluated the immune composition of the tumor and its microenvironment in patients with advanced/metastatic NSCLC who were treated with immunotherapy.

In the current study, the stringent definition of HPD (TGKr ≥2 and TTF ≤ 2 months) was applied and only NSCLC patients were included. The incidence of HPD (11.3%) was not much higher than previous literatures [7, 27, 29]. Several clinical factors were significantly associated with HPD. Among them, the patients carrying oncogenic driver mutation exhibited poor response to immunotherapy compared to those with no mutation. There are several possible explanations for this result. First, tumor mutational load in NSCLCs having oncogenic mutation is lower than that of wild type NSCLC [30–32]. Second, the TME of NSCLCs with oncogenic mutation is not usually inflamed, resulting in reduced interferon-γ signature [2, 32, 33]. Consistent with the report of Ferrara et al. [27], our data showed that HPD was significantly correlated with multiple metastatic sites (≥3).

Even if PD-L1 expression is not a prerequisite for anti–PD-1/PD-L1 inhibitors due to its variability and dynamicity, it has been widely used as standard predictive biomarker for immunotherapy [4]. Beyond PD-L1, tumor mutation burden, cytotoxic CD8+ T cells/TILs, an “immunoscore”, T cell receptor clonality, immune gene signature/RNA repertoire, and major histocompatibility complex class polymorphisms are being investigated [18, 19]. In the present study, tumoral PD-L1 expression tended to be lower in the HPD group compared to the PR/CR group and very low level of PD-L1 expression (< 1%) was one of the significant risk factors for HPD in univariate and multivariate analyses. Furthermore, we discovered three serologic markers (NLR, PLR, and CAR) at the first response assessment are independently correlated with HPD and particularly, CAR at the beginning of initial treatment and immunotherapy had still significant correlations. NLR and PLR, hematological indicators reflecting the changes of blood cell pattern, are a secondary local response to immunotherapy [21, 22, 34]. In contrast, CAR is an inflammatory indicator reflecting a patient’s general condition and cancer progression [35]. Several reports have noted that the serologic markers (NLR ≥ 5, PLR ≥ 150, and CAR ≥0.5) significantly correlated with HPD [22, 34]. In current study, CAR (≥ 0.5) before prior treatment as well as before and during immunotherapy, has predictive value for poor response to immunotherapy (HPD and PD). Inoue et al. reported that a high CAR index was associated with early death following the administration of immunotherapy [28]. There are some debates as to whether inflammation-based serologic markers obtained from systemic circulating blood denote the degree of immune response at the local tumor. For whatever reason, many studies strongly support the evidence that local immune response manifests adequate prognostic value [21]. The serologic markers, as well as imaging studies, may provide additional information concerning the tumor response to immunotherapy in patients with advanced NSCLC.

In the present study, the cells expressing T cell and TIL markers were abundant in the stromal region in disease-controlled group, whereas there were significantly more T regulatory cells co-expressing CD4+ and FOXP3+ in the entire tumoral and stromal area in poor response group. Previous studies have emphasized that pre-existing anti-tumor immunity and T cell exhaustion are associated with HPD [15, 16]. Moreover, Lo Russo et al. recently presented evidence that, upon immunotherapy-related Fc receptor engagement, tumor-associated macrophage reprogramming, plays a crucial role in HPD [29]. Our results presented the finding that M2-type macrophages and cancer-associated fibroblasts were widely distributed in the stromal region in the HPD group. This study provides the evidence that there is a close relation between HPD and M2-type macrophages throughout heterogeneous cellular components within the TME. Recent reports indicated that macrophages are influenced by the TME, causing them to adopt and facilitate epithelial–mesenchymal transition features, and transformed from M1 to M2 polarization, eventually resulting in rapid disease progression [2, 9, 10]. Of note, M1 and M2 signatures have important functional differences: M1 evinces an enhanced microbicidal and tumor resistant effect, while M2 plays a role in anti-parasite defense and immunoregulation [2, 36]. However, in human disease, their functional activities overlap and are far more dynamic. Notwithstanding, M2 macrophages are more dynamic than other immunologic indices and are regarded as poor prognostic biomarker for HPD.

NK cells are important cytotoxic, innate immune cells involved in the elimination of cancer cells [37]. There are two main NK cell subsets based on CD56 and CD16 expression: the CD56^bright^CD16 − NK subset produces abundant cytokines, including interferon-γ and tumor necrosis factor α, whereas the CD56^dim^CD16+ subset has high cytolytic activity and releases granules containing perforin and granzymes [38]. Several studies have discovered enhanced PD-1 expression on activated NK cells such as CD56 + ^bright^NK cells [37, 39]. We took note of the composition of CD56 + ^bright^CD16- NK and CD56^dim^CD16+ NK subsets in tumors and stroma. CD56 + ^bright^NK cells were remarkably increased in intratumoral region in the PR/CR group. Intratumoral PD-1+ NK cells are related to PD-L1 expressed on cancer cells and prevent the expansion and function of effector T cells and their exhaustion, eventually leading to immune evasion by the tumor. Based on these results, we assume that increased intratumoral CD56 + ^bright^NK cells may play an important role in the immunotherapy-initiated revitalization of T-cells in the PR/CR group.

The present study has several limitations. First, because this retrospective analysis was carried out for lung cancer patients who had multiple lines of treatment, the composition and function of immune cells in the tumor and the TME may be changed in response to cytotoxic chemotherapy compared to that of the initial diagnosis. Second, the amount of archival tissue available for this retrospective study was too insufficient to conduct a whole genome study such as next generation sequencing. Instead, we applied multiplex IHC to identify various immune cell markers. Third, owing to insufficient numbers in some subgroups based on tumor response pattern, it is difficult to draw widely applicable conclusions from the results.

HPD is a phenomenon caused by immunotherapy rather than by differences of treatment efficacy in biologically heterogeneous NSCLC patients [16, 29]. Our study indicates that some serologic indexes and the compositional changes of immune cells have meaningful associations with HPD in NSCLC patients receiving immunotherapy, but the analysis result may be stochastic. To better clarify this, we are conducting a prospective study to explore a mechanism underlying the development of HPD.

## Conclusion

HPD, a unique biologic process distinct from PD, is closely connected to short survival time. Our results suggest that to reduce risk of HPD by immunotherapy, clinical factors including heavy smoking, very low PD-L1 expression, multiple metastases, and a serological index, CAR should be fully considered beforehand. In addition, the composition of T-cell subsets, macrophages, and NK cells in the tumor and surrounding stroma may be useful to predict the tumor response to immunotherapy and aid in improving understanding regarding the dynamic and complex changes of immune cells.

## Supplementary Information

**Additional file 1: Supplementary Figure S1** CONSORT flow diagram for the present study. *HPD* hyperprogressive disease, *ICBs* Immune checkpoint blockades, *Non-HPD PD* non-HPD progressive disease, *NE* Not evaluable, *PR/CR* partial/complete response, *SD* stable disease.

**Additional file 2: Supplementary Figure S2** Spider plot depicting percentage change in the sum of the largest diameters of target lesions over time according to hyperprogressive disease status. *ICBs* Immune checkpoint blockades.

**Additional file 3: Supplementary Figure S3** Scatterplot of tumor response pattern and PD-L1 expression levels. Symbols (dots) in the scatterplot represent the tumoral PD-L1 (22C3) expressions. The mean level of PD-L1 expression in HPD group was significantly lower compared to that of SD/PR/CR group (*P* = 0.003). *HPD* hyperprogressive disease, *SD stable disease, PR/CR partial/complete response (TIF 881 kb)*

**Additional file 4: Supplementary Figure S4** Kaplan-Meier survival curve in each patient group according to the tumor response pattern (*n* = 211) ^†^. ^†^ Non evaluable group was excluded. *HPD* hyperprogressive disease, *Non-HPD PD* non-HPD progressive disease, *PR/CR* partial/complete response, *SD* stable disease.

**Additional file 5: Supplementary Table S1.** Clinical and pathologic characteristics (*N* = 231)

**Additional file 6: Supplementary Table S2.** Serologic inflammatory markers associated with HPD by univariate and multivariate analyses (*n* = 155)^† (DOCX 16 kb)^

## Data Availability

All data generated or analyzed during this study are included in this published article [and its supplementary information files.

## Abbreviations

ICBs: Immune checkpoint blockades
HPD: Hyperprogressive disease
NSCLC: Non-small cell lung cancer
TME: Tumor microenvironment
TILs: Tumor-infiltrating lymphocytes
NK: Natural killer
PD: Progressive disease
SD: Stable disease
PR/CR: Partial/complete response
LDH: Lactate dehydrogenase
NLR: Neutrophil-to-lymphocyte ratio
PLR: Platelet-to-lymphocyte ratio
CAR: C-reactive protein-to-albumin ratio
RECIST: Response Evaluation Criteria in Solid Tumors
TGKr: Tumor growth kinetics ratio
TTF: Time-to-treatment failure
IHC: Immunohistochemistry
NE: Non-evaluable
Treg cell: Regulatory T cell
EGFR: Epidermal growth factor receptor.
