# Supplementary figures and images for: The implications of clinical risk factors, CAR index, and compositional changes of immune cells on hyperprogressive disease in non-small cell lung cancer patients receiving immunotherapy
Source: BMC Cancer. 2021 Jan 5;21:19. doi: 10.1186/s12885-020-07727-y (PMC7786505; doi:10.1186/s12885-020-07727-y)

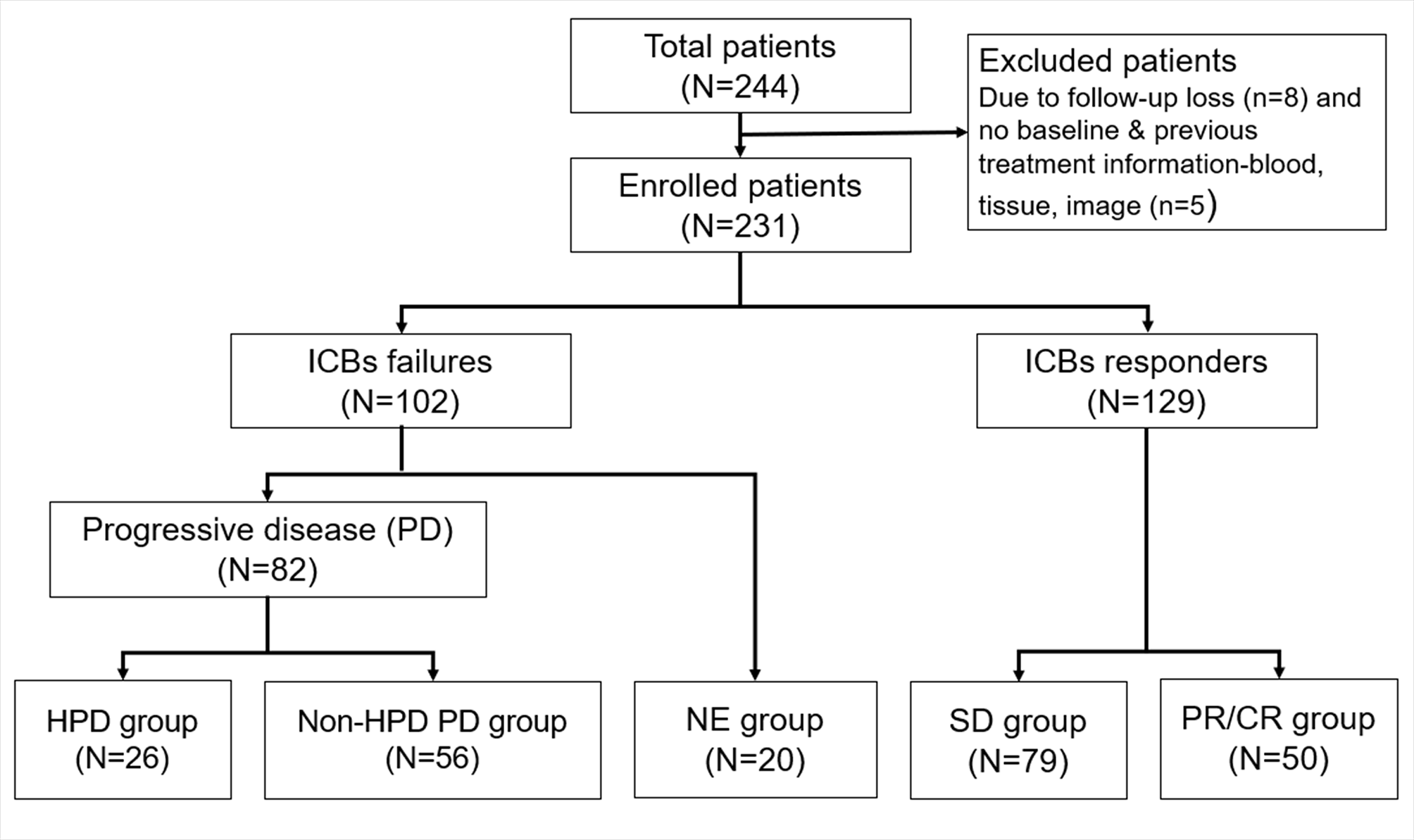

Supplement: Supplementary file 1 — Additional file 1: Supplementary Figure S1 CONSORT flow diagram for the present study. HPD hyperprogressive disease, ICBs Immune checkpoint blockades, Non-HPD PD non-HPD progressive disease, NE Not evaluable, PR/CR partial/complete response, SD stable disease. [file 12885_2020_7727_MOESM1_ESM.tif]

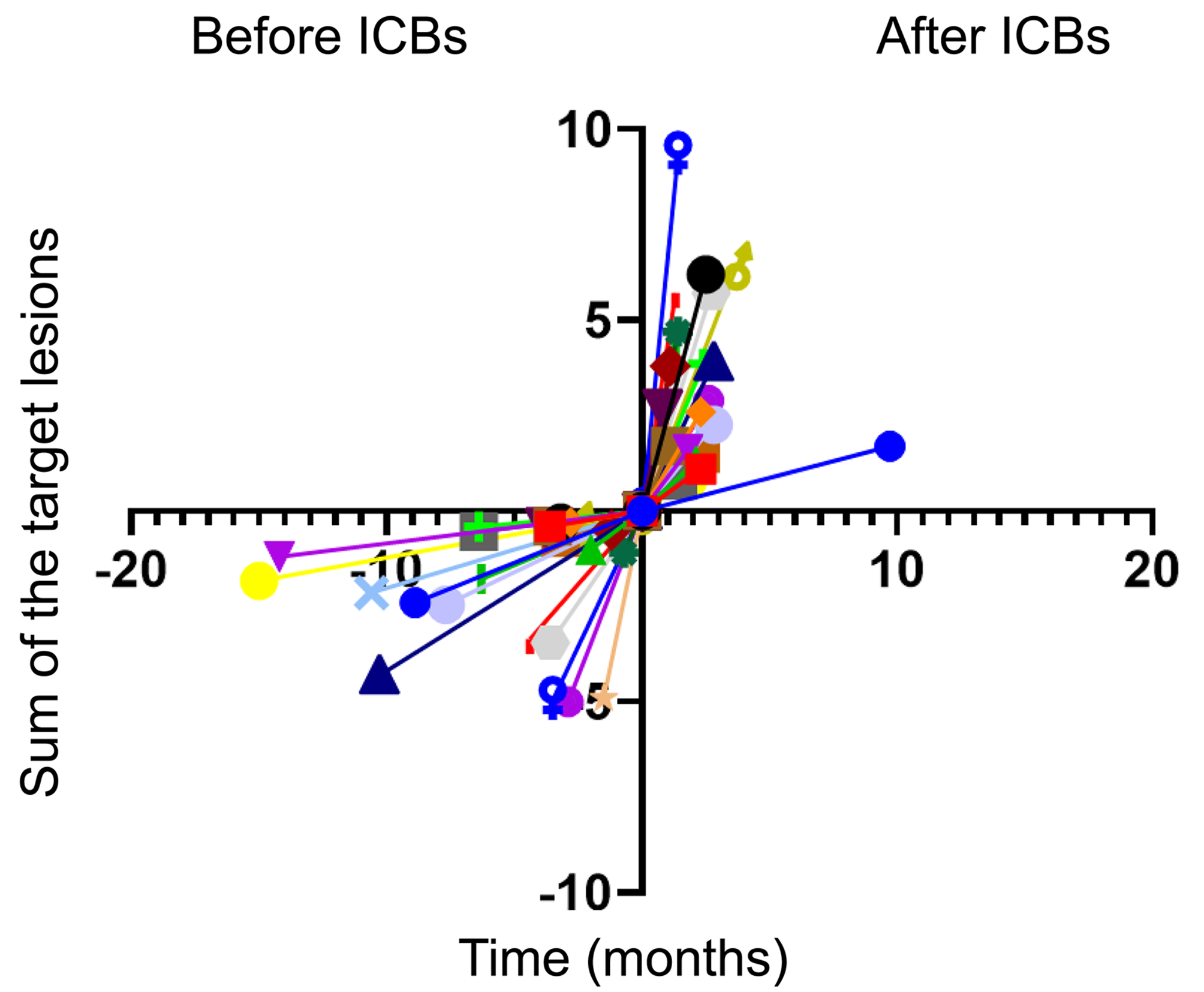

Supplement: Supplementary file 2 — Additional file 2: Supplementary Figure S2 Spider plot depicting percentage change in the sum of the largest diameters of target lesions over time according to hyperprogressive disease status. ICBs Immune checkpoint blockades. [file 12885_2020_7727_MOESM2_ESM.tif]

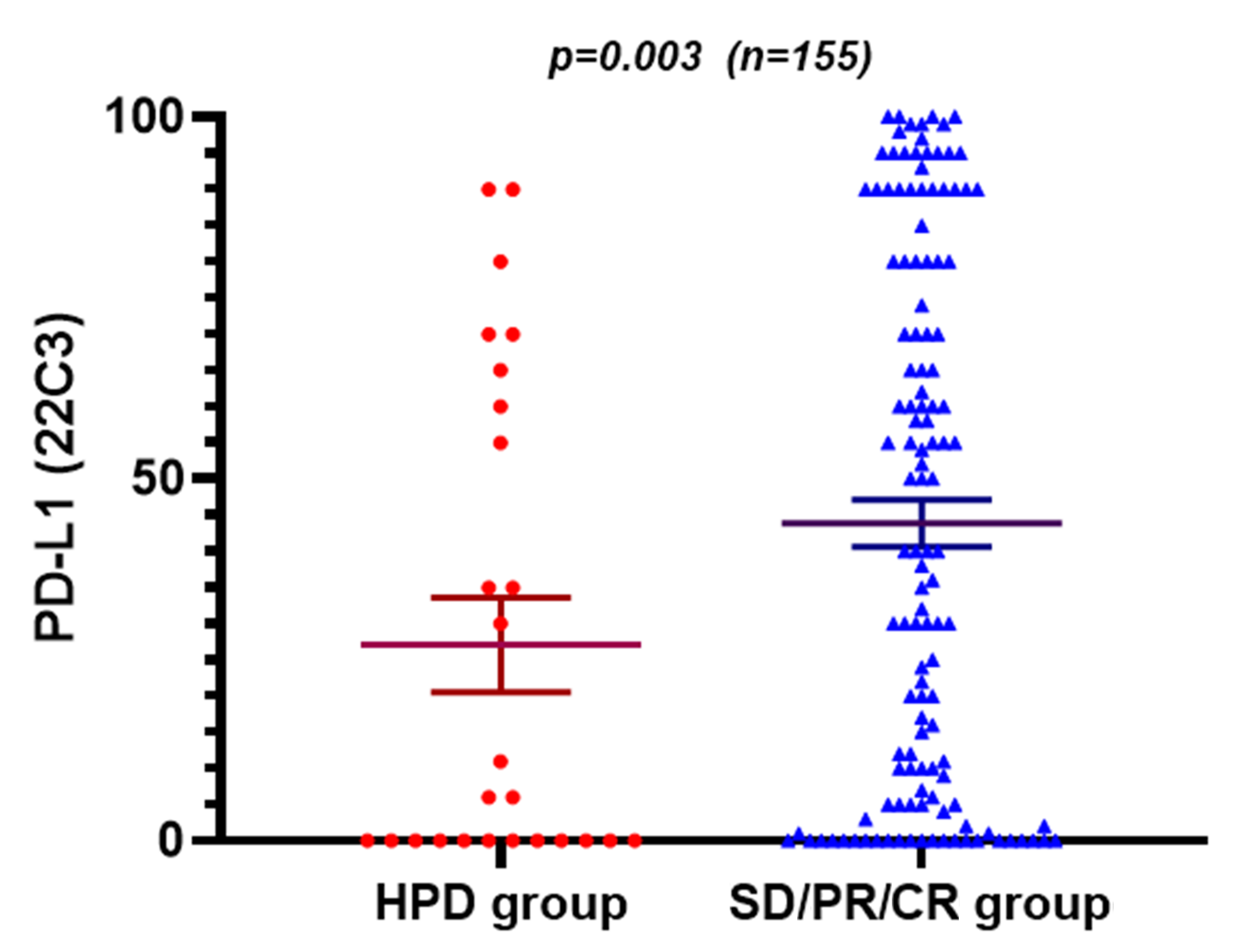

Supplement: Supplementary file 3 — Additional file 3: Supplementary Figure S3 Scatterplot of tumor response pattern and PD-L1 expression levels. Symbols (dots) in the scatterplot represent the tumoral PD-L1 (22C3) expressions. The mean level of PD-L1 expression in HPD group was significantly lower compared to that of SD/PR/CR group (P = 0.003). HPD hyperprogressive disease, SD stable disease, PR/CR partial/complete response (TIF 881 kb) [file 12885_2020_7727_MOESM3_ESM.tif]

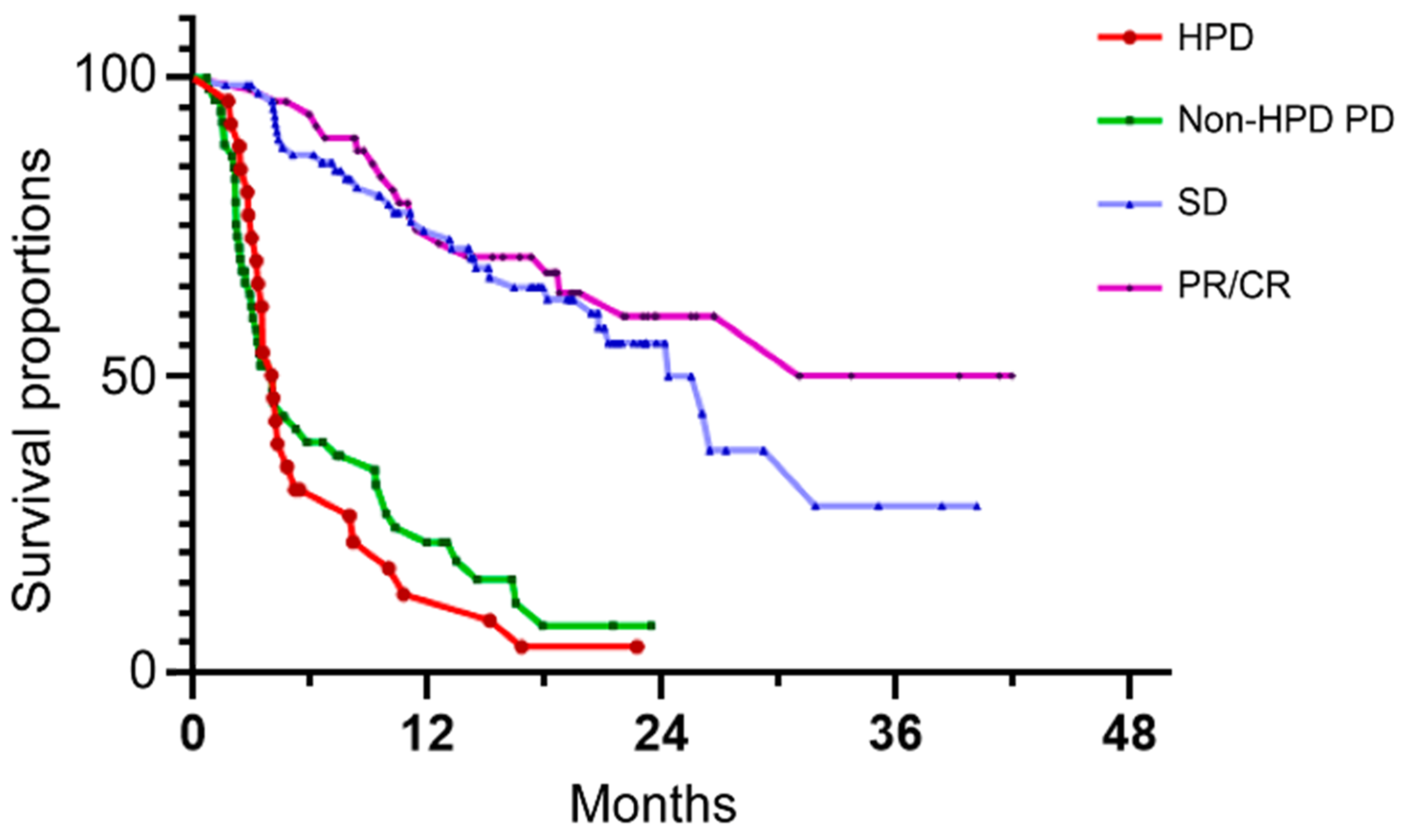

Supplement: Supplementary file 4 — Additional file 4: Supplementary Figure S4 Kaplan-Meier survival curve in each patient group according to the tumor response pattern (n = 211) †. † Non evaluable group was excluded. HPD hyperprogressive disease, Non-HPD PD non-HPD progressive disease, PR/CR partial/complete response, SD stable disease. [file 12885_2020_7727_MOESM4_ESM.tif]
