# Supplementary material for: The implications of clinical risk factors, CAR index, and compositional changes of immune cells on hyperprogressive disease in non-small cell lung cancer patients receiving immunotherapy
Source: BMC Cancer. 2021 Jan 5;21:19. doi: 10.1186/s12885-020-07727-y (PMC7786505; doi:10.1186/s12885-020-07727-y)
Supplement: Supplementary file 5 — Additional file 5: Supplementary Table S1. Clinical and pathologic characteristics (N = 231) [file 12885_2020_7727_MOESM5_ESM.docx]

**Supplementary Table 1.** Clinical and pathologic characteristics (N = 231)

| Characteristics | Data |
| --- | --- |
| Age (y) | 64.2 (29-92) |
| Sex |  |
| Men | 173 (74.9) |
| Women | 58 (25.1) |
| ECOG Performance status |  |
| 0 | 162 (70.1) |
| 1 | 63 (27.3) |
| 2 | 6 (2.6) |
| Smoking history |  |
| Never smoker | 69 (29.9) |
| Current smoker | 57 (24.7) |
| Ex-smoker | 105 (45.5) |
| Smoking (pack*year) | 162 |
| <20 | 18 (11.1) |
| ≥20 (heavy smoker) | 144 (88.9) |
| Clinical stage at diagnosis |  |
| ≤Stage IIIc | 76 (33.6) |
| Stage IVa | 93 (36.4) |
| Stage IVb | 62 (31.8) |
| Histology |  |
| Adenocarcinoma | 162 (70.1) |
| Squamous cell carcinoma | 62 (26.8) |
| Others (Adenosquamous cell carcinoma, etc.) | 7 (3.1) |
| Metastatic site |  |
| Lymph node (SCN or extrathoracic) | 89 (38.5) |
| Pleural metastasis | 80 (34.6) |
| Malignant pleural/pericardial effusion | 60 (26.0) |
| Lung to lung metastasis | 57 (24.7) |
| Bone metastasis | 76 (32.9) |
| Brain or CNS | 47 (20.3) |
| Liver | 28 (12.1) |
| Miscellaneous^†^ | 18 (7.8) |
| Number of metastatic sites |  |
| 0 | 13 (5.6) |
| 1 | 89 (38.5) |
| 2 | 63 (27.3) |
| 3 | 37 (16.0) |
| ≥4 | 29 (12.6) |
| Oncogenic driver mutation (n=43) |  |
| EGFR | 31 (13.4) |
| ALK / ROS-1 | 4 (1.7) |
| Others (K-Ras, B-RAF, BRCA…) | 8 (3.4) |
| Immune checkpoint blockade |  |
| Pembrolizumab | 98 (42.4) |
| Nivolumab | 108 (46.8) |
| Durvalumab | 10 (4.3) |
| Avelumab | 6 (2.6) |
| Atezolizumab | 2 (0.9) |
| Switch to other ICB or combination | 7 (3.0) |
| Number of prior systemic treatment |  |
| ≤1 | 141 (61.0) |
| 2 | 53 (22.9) |
| 3 | 17 (7.4) |
| ≥4 | 20 (8.7) |
| Tumor response to ICBs |  |
| CR/PR | 50 (21.6) |
| SD | 79 (34.2) |
| PD | 82 (35.5) |
| UnKnown | 20 (8.7) |

Values are presented as mean (range) or number (%).

^†^Miscellany includes lymphagitic metastasis and metastasis to the adrenal glands, skin, kidney, bowel.
