# Supplementary material for: The implications of clinical risk factors, CAR index, and compositional changes of immune cells on hyperprogressive disease in non-small cell lung cancer patients receiving immunotherapy
Source: BMC Cancer. 2021 Jan 5;21:19. doi: 10.1186/s12885-020-07727-y (PMC7786505; doi:10.1186/s12885-020-07727-y)
Supplement: Supplementary file 6 — Additional file 6: Supplementary Table S2. Serologic inflammatory markers associated with HPD by univariate and multivariate analyses (n = 155)† (DOCX 16 kb) [file 12885_2020_7727_MOESM6_ESM.docx]

**Supplementary Table 2.** Serologic inflammatory markers associated with HPD by univariate and multivariate analyses (n = 155)^†^

|  | **Univariate** | | **Multivariate** | |
| --- | --- | --- | --- | --- |
|  | **HR (95% CI)** | ***P*-value** | **HR (95% CI)** | ***P*-value** |
| NLR (base^‡^), ≥5 vs <5 | 2.20 (0.88-5.52) | 0.092 | 1.53 (0.55-4.26) | 0.4117 |
| PLR (base), ≥150 vs <150 | 1.67 (0.68-4.13) | 0.2644 | 1.24 (0.47-3.31) | 0.6638 |
| CAR (base), ≥0.5 vs <0.5 | 2.62 (1.09-6.33) | **0.0318** | 2.18 (0.85-5.55) | 0.1031 |
| LDH (base), ≥400 vs <400 | 0.35 (0.09-1.36) | 0.1290 | 0.77 (0.32-1.85) | 0.553 |

^†^ Non evaluable group and Non-HPD PD group were excluded.

^‡^ Base implies at the beginning of immunotherapy

*HPD* hyperprogressive disease, *HR* hazard ratio, *CAR* C-reactive protein-albumin ratio, *ICB* immune checkpoint blockades, *LDH* lactate dehydrogenase, *NLR* neutrophil-to-lymphocyte ratio, *PLR* platelet-to-lymphocyte ratio
